# Modeling public attention behavior on badminton’s ecosystem evolution via complex network theory

**DOI:** 10.3389/fspor.2025.1647207

**Published:** 2025-11-07

**Authors:** Yongjie Liang, Yuan Xu, Runsheng Gu, Jun Hu, Huijia Li

**Affiliations:** 1 College of Physical Education, Guizhou University of Finance and Economics, Guiyang, China; 2 School of Statistics, Nankai University, Tianjin, China; 3 School of Economics and Management, Inner Mongolia University, Hohhot, China

**Keywords:** visibility graph, badminton venues, evolutionary dynamics, complex system, complex network

## Abstract

The growing convenience of badminton and the expansion of related venues have increased the complexity of public attention toward the sport, making nonlinear research methods an effective approach for exploring the evolutionary dynamics of its popularity. The visibility graph algorithm was adopted to characterize badminton search volume as a dynamic behavioral indicator of the badminton attention complex system, enabling systematic investigation of the system's evolutionary process and dynamical characteristics. Results show that the evolutionary process of the badminton attention complex system exhibits small-world and approximately scale-free network properties, coupled with chaotic dynamical behavior characteristics. This indicates that the badminton attention complex system is ultimately a deterministic nonlinear dynamical system governed by chaotic dynamics.

## 1 Introduction

In recent years, with the rapid development of the global sports industry, badminton, as a globally participated sport, has demonstrated significant spatial heterogeneity and complex evolutionary characteristics. According to the latest BWF data (1), the Asian region accounts for 82% of global tournament viewership, with China’s Thomas & Uber Cup events contributing 35% . The Indian market has shown particularly remarkable growth, with a 217% increase in viewership for the 2024 Open compared to 2019. Meanwhile, Europe has developed an elite training model represented by Denmark, while North America promotes commercialization through professional leagues. This regional differentiation indicates that the evolution of badminton’s popularity is a complex nonlinear process influenced by multiple factors, including the digital economy, cultural dissemination, and policy interventions.

Traditional research on sports viewership has predominantly employed linear causal models (2, 3). However, the evolution of badminton’s popularity in the digital era demonstrates characteristic features of a Complex Adaptive System (CAS): social media dissemination creates mutation effects, as evidenced by Viktor Axelsen’s 2021 YouTube training videos triggering a 43% surge in badminton equipment sales in Denmark, while Asia’s technical prowess and Europe’s organizational innovations collectively shape the global competitive landscape of badminton (4). The CAS theory provides a novel framework for understanding such nonlinear dynamics (5, 6). Proposed by Holland, CAS theory posits that systems consist of autonomous decision-making agents that adapt their behavioral strategies through environmental feedback, thereby driving systemic evolution (6). In sports viewership research, the strategic evolution of deep reinforcement learning agents (7) and behavioral pattern adjustments of financial market investors (8) offer analogous cases to the complex interactions among media, fans, and athletes. This observed nonlinear growth in badminton’s popularity aligns with CAS theoretical predictions (9).

Self-organization theory further explains the emergence of order in this process without central control (10). Drawing on Prigogine’s dissipative structure theory, both the dynamic equilibrium of information entropy in online communities (11) and threshold effects in technological innovation diffusion (12) demonstrate self-organizing characteristics in badminton’s popularity evolution. The formation of regional engagement disparities exhibits distinct path dependence, as evidenced by China’s “pyramid-style” development model contrasting sharply with Europe’s “club-based” system (13–15). However, current research faces critical limitations: traditional linear models cannot adequately capture social media-induced mutation effects in the digital era; most studies remain confined to macro-level national analyses; and few have effectively integrated digital footprint data to reveal dynamic engagement processes (16–18).

With developing an analytical framework integrating Complex Adaptive Systems (CAS) theory and self-organization theory, drawing upon multiple theoretical foundations including Moore’s Law regarding technological exponential growth (19), Scheffer’s critical transition theory (14), and Batty’s research on urban emergence (20) to explain badminton’s nonlinear popularity evolution. Technological parallels emerge between semiconductor innovation [e.g., 3D chip architecture’s adaptive progression (21)] and badminton equipment’s “technology-market” co-evolution, exemplified by Victor’s tournament co-branding strategy driving 12% premium racket sales growth in 2023 (15). Social system analyses reveal consistent dynamics between badminton’s popularity propagation and three phenomena: urban spatial aggregation (20), social media’s uneven information diffusion (16), and algorithmic recommendation feedback loops (17). Empirical evidence demonstrates economic correlations [1.3% badminton consumption increase per 1% GDP growth (22)] and tournament spillover effects [Guangzhou’s 20% venue increase post-2002 Thomas/Uber Cups (14)], collectively illustrating the sport’s complex systemic interactions.

Making significant theoretical contributions by employing the Complex Adaptive Systems (CAS) framework to reveal the nonlinear characteristics and driving mechanisms behind badminton’s popularity evolution, proposing the novel concept of “digitally-driven critical moments” to address theoretical gaps in traditional sports viewership research within the digital communication era. Practically, the findings provide scientific foundations for regional badminton promotion strategies, as evidenced by Xie et al.’s (18) demonstration of new measurement approaches (social media heat analysis and web search indices) effectively predicting popularity fluctuations, with Abd Shukor and Md Sharif (23) further validating the efficacy of social media data in forecasting youth participation rates.

The paper focuses on three core research questions: (1) the dynamic mechanisms and driving factors behind badminton’s popularity evolution, (2) regional variations in its developmental trajectories, and (3) the construction of effective measurement models in the digital era. Methodologically, it adopts a multi-dimensional approach: theoretically integrating complex systems frameworks to examine economic factors, media dissemination, and tournament systems (1, 24, 25); empirically employing multi-source data analysis that combines traditional metrics [event viewership (26, 27), participation scale (23), and consumer market size (28)] with emerging indicators [social media engagement (18)]; and practically applying insights from Bastug et al.’s (29) research on athletes’ cognitive capabilities and Habibi’s (30) social network analysis of badminton communities to explore the relationship between sports participation and social capital accumulation.

This study demonstrates innovation by incorporating digital-era characteristics into a Complex Adaptive Systems (CAS) theoretical framework to construct dynamic models, proposing a “critical moment identification” methodology, and establishing a multidimensional analytical framework integrating traditional data with digital footprints. However, challenges persist: current modeling approaches often oversimplify higher-order interaction variables (31), the applicability of CAS theory to sports sociology’s unique contexts requires further validation (32), and empirical studies face limitations in sample representativeness and cultural dimensionality (33–35). Future research should emphasize cross-cultural comparisons, develop more precise popularity measurement methods, and explore applications of AI-assisted training systems (36–38) along with sustainable technologies like biodegradable shuttlecocks (39–41). As a paradigmatic case of complex systems, research on badminton’s popularity evolution not only enriches sports sociology theory but also provides scientific foundations for formulating region-specific sports development policies (42), advancing the discipline’s innovation in the data-driven era.

## 2 Theoretical framework and methodological approach

### 2.1 Badminton attention complex system

Dynamic Systems Theory (DST) is a theoretical framework at the intersection of cognitive science, developmental psychology, ecology, and complex systems science. It advocates understanding cognition, behavior, and social phenomena from the perspectives of temporal evolution and nonlinear interactions. Its core proposition is that cognition is not the product of static representations but rather a self-organizing process emerging from the dynamic interactions among the brain, body, and environment.

Public attention in badminton refers to the collective search behavior patterns related to badminton observed on social media platforms. The complex system of badminton attention can be conceptualized as an interconnected network comprising multiple interacting nodes. Through clustering similar temporal patterns from measurable system variables, we can identify distinct community structures within the network. These communities, also referred to as modules or functional groups, represent subsets of network nodes that exhibit denser internal connections compared to other network components (43).

In temporal networks constructed from social media search data using “badminton” as the key term, nodes within the same community typically demonstrate synchronized attention cycles. From the perspective of complex system evolution, these communities correspond to distinct developmental phases of public engagement with badminton (44). Critical transition nodes serving as boundaries between communities act as mediating hubs in the network. These tipping points signify breakthrough moments in the measurable time-series data of the badminton attention system, marking both the conclusion of a previous developmental phase and the initiation of a new evolutionary stage. Characterized by extensive connectivity with multiple community nodes, these pivotal points exert substantial influence on neighboring node behaviors and play a crucial role in system dynamics reconstruction (45). They represent significant temporal markers in the evolutionary trajectory of public attention toward badminton.

Under nonlinear interactions, the badminton attention complex system may transition from a completely ordered and stable phase to a highly unstable and unpredictable chaotic state (46). Chaos represents a deterministic yet seemingly irregular motion pattern inherent in nonlinear dynamical systems, constituting neither pure disorder nor simple order, but rather an organized disorder—a sophisticated ordered state (47). Characterized by inherent stochasticity within deterministic structures, it embodies the unity of order and disorder, stability and instability, marking new evolutionary directions for ordered systems.

When the badminton attention complex system approaches the critical threshold where order and chaos intersect—the domain Langton conceptualized as the “edge of chaos”—it enters a transitional zone described as “the boundary of chaos” or “the onset of chaos” (46). This liminal space, where novel phenomena emerge through system-wide “emergence”, represents the most dynamic and innovative evolutionary phase. Here, existing system properties and structures gradually disintegrate, while new attributes and configurations emerge through holistic interactions, enabling the system to ascend to higher organizational complexity.

Should the system exceed the chaos boundary and manifest disordered behavior, implementing chaos control strategies becomes essential. This involves modulating critical parameters driving system instability to facilitate transition from chaotic disorder to ordered states (48). Agents engaged in badminton-related search activities must maintain sufficient dynamic adaptability and proactive agency, formulating effective decision-making protocols that align with system self-organization tendencies to foster new stable configurations.

Employing nonlinear methodologies to investigate the evolutionary dynamics of the badminton attention complex system enables researchers to harness its self-organizing properties and adaptive nonlinear interactions. Such approaches facilitate the creation of favorable evolutionary environments while providing essential foundations for scientific prediction and regulation of human behavioral patterns in this context.

### 2.2 Chaos theory

The structure of time series is conserved in the topology of mapped network graphs, with specific correspondences as follows:

Periodic time series map to regular network graphs, and their degree distribution follows a discrete distribution. Fractal time series map to scale-free network graphs, and their degree distribution obeys a power-law distribution. Both random time series and chaotic time series can be mapped to horizontal visibility graphs with exponential degree distribution. This means the node degree distribution of the graph follows an exponential distribution, where the cumulative distribution appears approximately as a straight line in a semi-logarithmic coordinate system. The degree distribution of the graph is expressed by the formula as shown in Equation 1:

$$P(k)=e^{-\lambda k}$$ (1)

In this formula, k represents the degree, and λ denotes the rate parameter.

The value

$$\lambda_{c}=\ln\;(2/3)=0.405$$ (2)

acts as an effective boundary between random and chaotic processes, separating the stable state from the chaotic state of the system. Specifically in the Equation 2:

- When $\lambda <\lambda_{c}$, the system exhibits a chaotic process, which is unstable and difficult to predict.
- When $\lambda >\lambda_{c}$, the system corresponds to a correlated random process, characterized by stability, orderliness, and high predictability.
- When $\lambda =\lambda_{c}$, the system corresponds to an uncorrelated random process (49).

Although random processes and chaotic processes share numerous common characteristics and have minimal differences, they have a core distinction. Chaotic processes possess finite-dimensional attractors—these attractors can be reconstructed and exhibit “intrinsic randomness”—and their time series are generated by deterministic nonlinear dynamic systems. In contrast, random processes originate from infinite-dimensional attractors, and their time series are generated by random dynamic systems (49).

Furthermore, the horizontal visibility graph algorithm shows robustness and reliability in the detection of chaotic and random time series. Compared with other commonly used methods, it has superior detection performance for chaotic time series, random time series, and noisy systems (50).

### 2.3 Horizontal visibility graph

**Figure 1:**
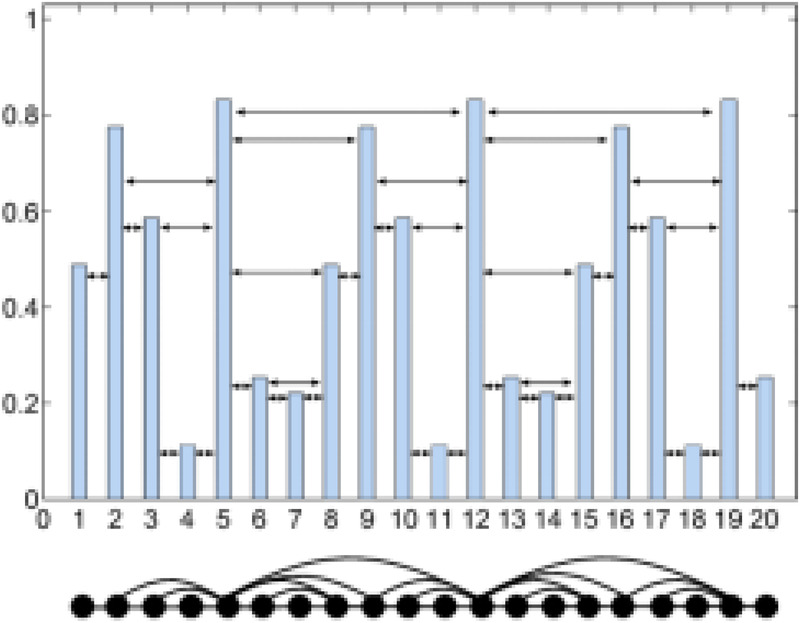
Horizontal visibility graph.

Horizontal Visibility Graph (HVG) (51) is an algorithm that transforms time series data into a graph structure, enabling the analysis of sequence complexity and structural features. The formal definition and construction process are outlined as follows:

Consider a time series $\left\{x_{1},x_{2},\ldots ,x_{N}\right\}$ of length N (e.g., financial prices, meteorological measurements). Node Generation: Each data point $\left(x_{i}\right)$ in the series is mapped to a corresponding node $v_{i}$ in the graph. Edge Connection Rule: For any two nodes $v_{i}$ and $v_{j}$ with (i<j), an undirected edge is established if and only if the following horizontal visibility criterion is satisfied: Horizontal Visibility: All intermediate data points $x_{k}$, where (i<k<j) between $x_{i}$ and $x_{j}$ must obey $x_{k}<\min(x_{i},x_{j})$. This ensures that no “obstacle” (i.e., a higher or equal value) blocks the horizontal line of sight between $x_{i}$ and $x_{j}$. as shown in the Figure 1 and Equation 3.

$$(v_{i},v_{j})\in E\;⟺\;x_{k}<\min(x_{i},x_{j}),\;\forall k\in (i,j).$$ (3)

The structural properties of time series are conserved in their mapping to network topologies: Periodic time series map to regular networks with discrete degree distributions (e.g., uniform node degrees). Fractal time series map to scale-free networks exhibiting power-law degree distributions $P(k)∼k^{-\gamma }$ . Stochastic/chaotic time series map to Horizontal Visibility Graph (HVG) networks with exponential degree distributions, where the node degree distribution follows $P(k)=e^{-\lambda k},$ where k: degree, λ: rate parameter. The critical threshold $\lambda_{c}=\ln\;(2/3)\approx 0.405$ acts as a phase boundary: Chaotic regime $\lambda <\lambda_{c}$: Unstable dynamics with poor predictability Ordered regime $\lambda >\lambda_{c}$: Stable correlated stochastic processes Critical state $\lambda =\lambda_{c}$: Memoryless white noise.

### 2.4 Stage recognition model

Given a discrete time series data set $Z(t)=(z_{1}(t),z_{2}(t),\ldots ,z_{k}(t))$, t=1,2,…,T, denotes the duration of this discrete time series, assuming that the time series can be divided into N(1<N≤T) phases, is the time point of division.Thus the results of the time series phasing satisfy the following requirements:

1. $t_{i}$ satisfy $0=t_{0}<t_{1}<t_{2}<\cdots <t_{N}=T$;
2. The corresponding phases are $[t_{0}+1,t_{1}],$ $\left[t_{1}+1,t_{2}],\ldots ,[t_{N_{1}}+1,t_{N}\right]$, respectively, and we can also refer to these phases as the N phases of Z(t).

Time series data phase segmentation means that a mathematical model is used to divide the whole set into several consecutive subsets, each of which is a phase. By analyzing different phases, the features of different phases are better extracted.

Barabasi and Albert first proposed to use the modularity degree Q as an objective function to recognize the association structure in a network . After a large amount of real data and experimental results, it was shown that nodes and edges describe the constituent units of complex systems and their interactions, respectively. In the study of complex networks, it is found that the classification of nodes in the network is highly correlated with the structure of the network, and one of the important manifestations of this is that the connecting edges between nodes in the same category are very close, and the connecting edges between nodes that are not in the same category are very sparse, and therefore nodes can be categorized based on these features obtained in the network. Thus the clustering analysis of nodes in a network can be converted into an optimization problem for the optimal classification of the connecting edge relationships within the network. Its modularity Q, as one of the commonly used objective functions, has been widely used in association delineation in the following functional form:

$$Q=\sum_{i}^{k}\left[\frac{l_{i}^{\mathrm{in}}}{L}-{\left(\frac{d_{i}}{2L}\right)}^{2}\right].$$ (4)

In order to design an efficient objective function to measure the goodness of our partitioning results, we make a substitution of this modularity function. Since a viewable graph network is an undirected graph network and has no edges connected to itself, i.e., A(i,j)=A(j,i) and.A(i,i)=0.Then for a given a network G(V,E), V is the set of nodes, E is the set of edges, and N=|V| and M=|E| denote the number of nodes and edges respectively. Assume that G(V,E) is an undirected network without self-loops. If g(v,e) is a clustering of G(V,E) and n=|v| and m=|e|, the ratio of the intra-cluster edges (i.e., edges whose both ends lie inside the clustering g(v,e)) can be written as in Equation 5:

$$\ell_{\mathrm{in}}=\frac{2m}{n(n-1)}.$$ (5)

Similarly the ratio of inter-cluster edges (i.e., edges connecting the clusters g(v, e) to the rest of the network) can be obtained in Equation 6:

$$\ell_{\mathrm{in}}=\frac{m_{e\times t}}{n(n-1)},$$ (6)

where $m_{e\times t}$ denotes the number of edges in the cluster.

According to the definition of the clustering structure, it is required that the density of the intra-cluster edges is greater than the density of the inter-cluster edges, i.e., $\ell_{\mathrm{in}}>\ell_{\mathrm{out}}$. Also the average side ratio of the network G(V,E) is defined asThen the ratio of intra-group edges will be greater than the average and inter-group edges. This relationship can be expressed by the following in Equation 7

$$\ell_{\mathrm{in}}>\ell_{\mathrm{rand}}>\ell_{\mathrm{out}}.$$ (7)

Next, assume that the network G(V,E) can be partitioned into c network clusters, i.e., $g_{1},g_{2},\ldots ,g_{c}$ , whose number of points and edges are $n_{1},n_{2},\ldots ,n_{c}$ and $m_{1},m_{2},\ldots ,m_{c}$ , Letting be the proportion of cluster interiors in the null model, Equation 4 can be organized as in Equation 8:

$$\ell_{\mathrm{in}}(q)=\frac{m_{q}}{\frac{n_{q}(n_{q}-1)}{2}}>P\Rightarrow 2m_{q}>P_{n_{q}}(n_{q}-1).$$ (8)

We take Q as the objective function and the decision variable is the length of each segment $\{x_{1},x_{2},\ldots \cdots ,x_{n}\},k$ is the length of time its modeled as follows:

$$\begin{array}{cc} \max Q\\ s.t. & \sum_{\;j=1}^{n}t_{i}=T\\ & 0\leq t_{i}\leq T \end{array}$$ (9)

In this model as shown in Equation 9, the sum of all segment lengths is equal to the length of the time series, and the length of each segment is greater than or equal to 0 less than or equal to k, that is to say, change the time series to be divided into at least one segment.

## 3 Data

**Figure 2:**
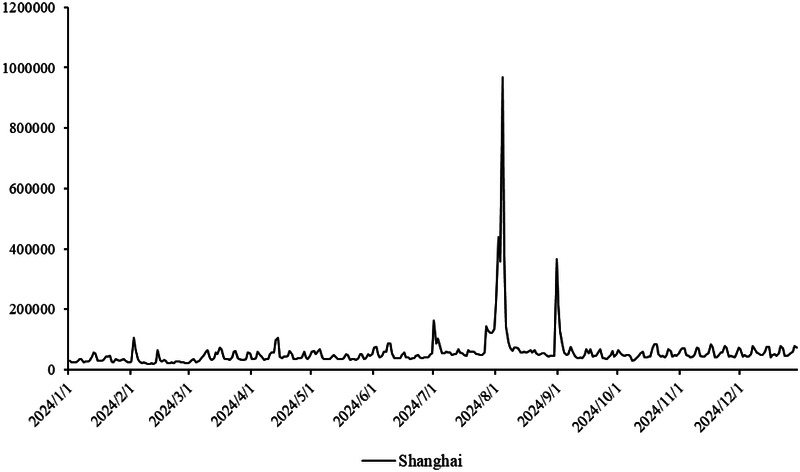
Search frequency data of Shanghai.

**Figure 3:**
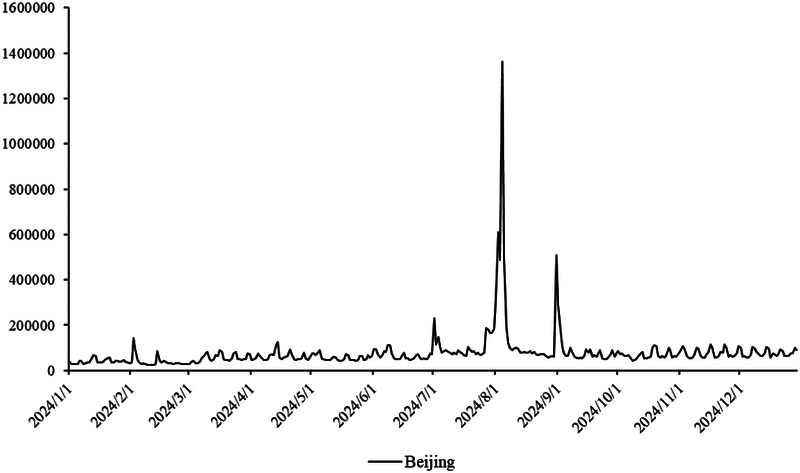
Search frequency data of Beijing.

**Figure 4:**
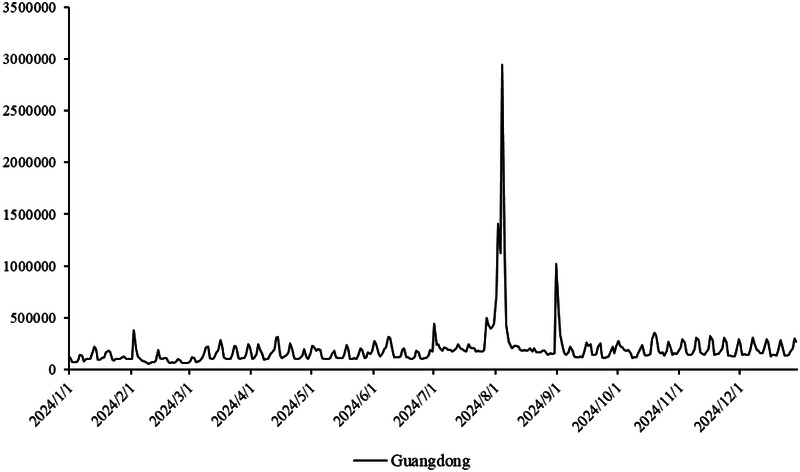
Search frequency data of Guangdong.

**Figure 5:**
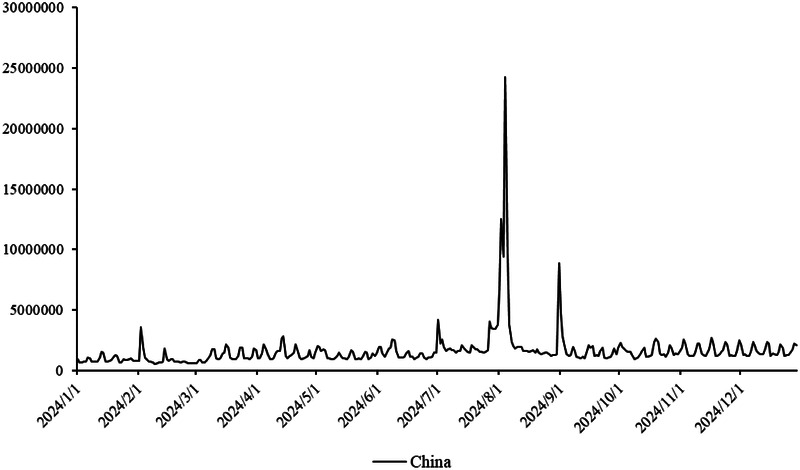
Search frequency data of China.

The primary dataset consists of daily search volume counts for the keyword “badminton” on Douyin (the Chinese counterpart of TikTok), collected via the Douyin Open Platform API (v2.0) with official data access authorization. The data covers four geographic units to capture regional and national dynamics, We collected search frequency data from Beijing, Shanghai, Guangdong and China spanning January 2024 to December 2024. As illustrated in Figure 3, the x-axis represents time (by date), while the y-axis denotes the daily search volume, as shown in Figures 2–5.

Provincial-level: Beijing (northern China), Shanghai (eastern China), Guangdong (southern China)—three regions with distinct badminton development models (e.g., Beijing’s policy-driven promotion, Shanghai’s commercialized sports ecosystem, Guangdong’s grassroots community engagement).

National-level: Aggregated daily search volume across all Chinese provinces, reflecting macro-level attention trends.

Time span: January 1, 2024, to December 31, 2024 (366 days, including February 29, 2024, a leap year), ensuring coverage of key events influencing badminton attention (e.g., 2024 Paris Olympics, July 26–August 11; National Fitness Day, August 8).

## 4 Experiment and result

**Figure 6:**
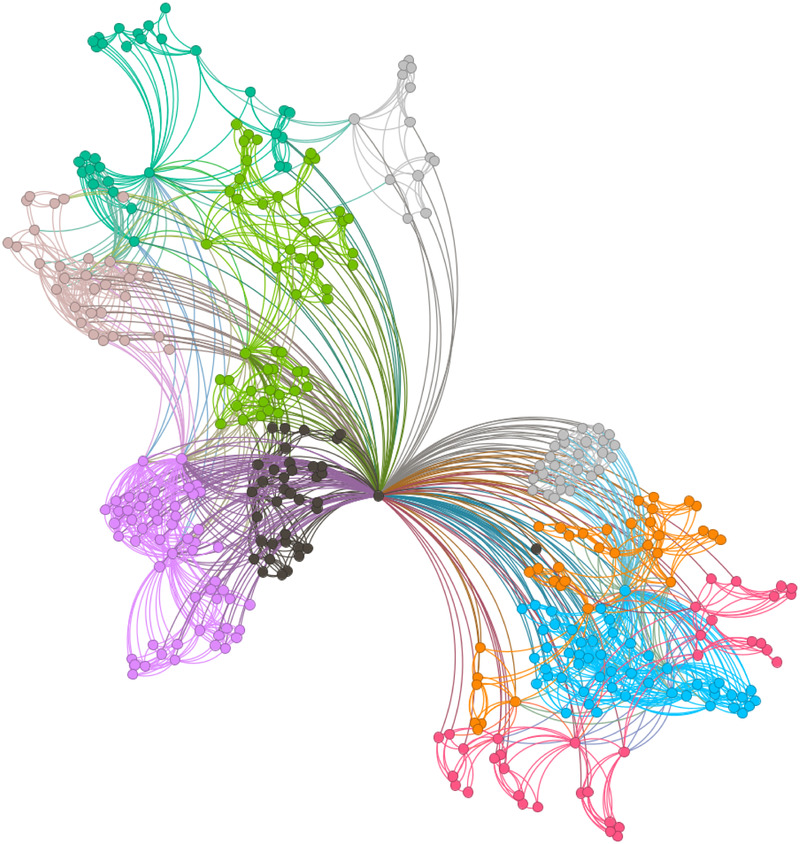
Visbility graph of Shanghai.

**Figure 7:**
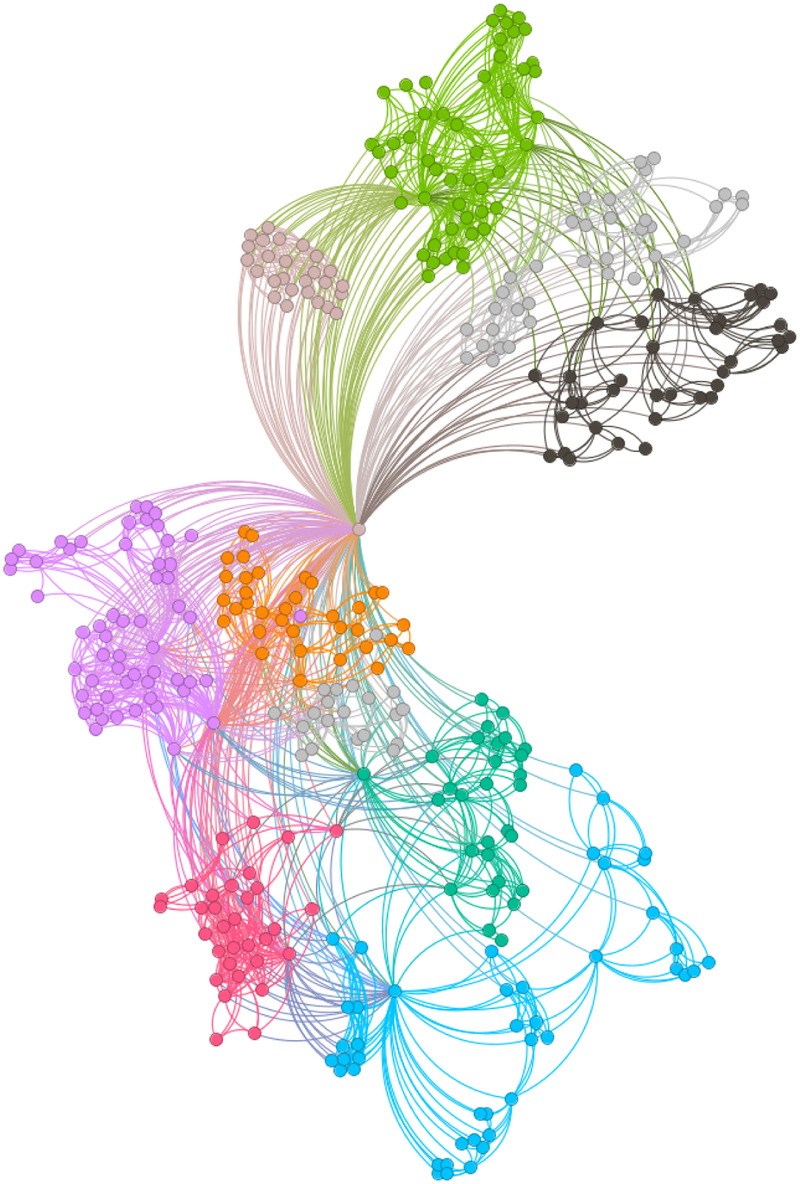
Visbility graph of Beijing.

**Figure 8:**
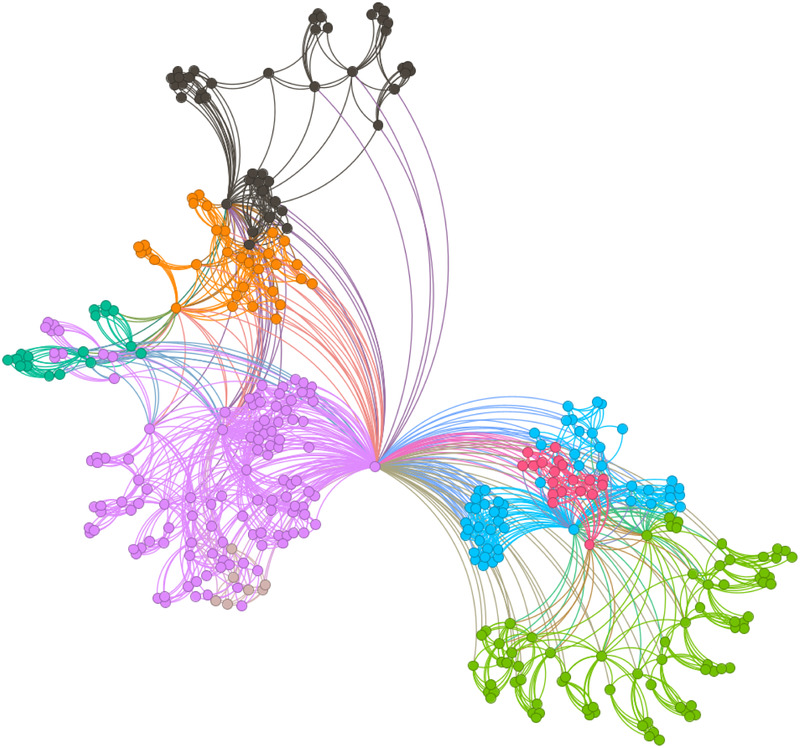
Visbility graph of Guangdong.

**Figure 9:**
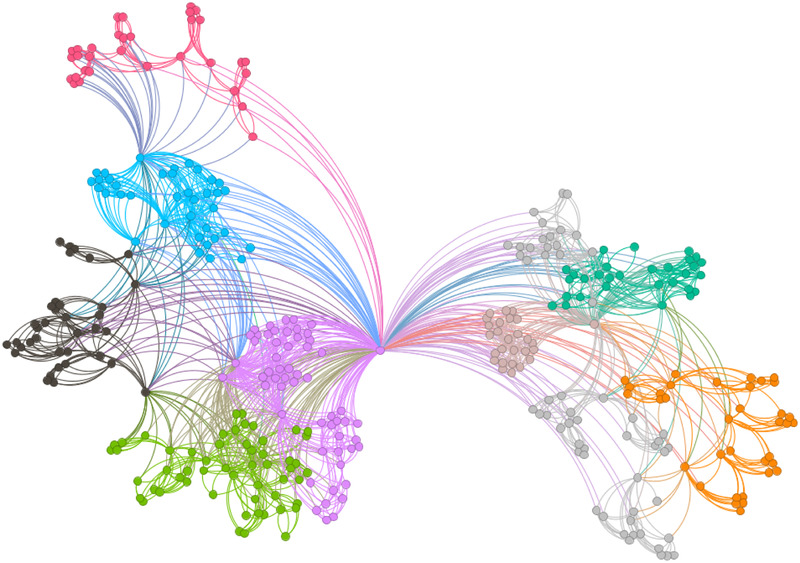
Visbility graph of China.

Figures 6–9 illustrate the evolution of badminton attention across Shanghai, Beijing, Guangdong, and China as a whole, revealing its manifestation as a Complex Adaptive System (CAS) under varying regional contexts.

The network structure in Shanghai demonstrates a high degree of clustering and cohesion, indicating that the dissemination of badminton attention is not solely dominated by a few central nodes. Instead, these nodes, through tight internal connections, rapidly influence a broader population. Shanghai’s small-world effect and scale-free properties undoubtedly reflect the efficiency of information transmission and the high connectivity of its social structure. Specifically, the degree distribution in Shanghai follows a power-law, illustrating the dominant role of a few nodes in driving network expansion. Moreover, during major badminton events or market activities, the surge in attention manifests as a sudden network behavior, representing the characteristic shift from “ordered” to “disordered” states inherent in complex systems. In other words, Shanghai’s attention network continuously oscillates between stability and fluctuation, displaying a dynamic process of aggregation and expansion.

The network structure in Beijing, in contrast, exhibits greater decentralization with lower connectivity between nodes, suggesting a more balanced distribution of badminton attention among the public. This network structure is closely related to Beijing’s cultural context and social environment. As the capital, Beijing’s social groups are more diverse, with badminton attention influenced both by government promotional policies and by the impact of diverse cultural activities. At this stage, Beijing’s network shows characteristics of polycentricity, meaning that the expansion of information relies not only on the concentration effect of a few nodes but also on the broad participation and interaction of diverse groups to drive the evolution of the entire network. In this context, Beijing’s badminton attention network displays strong periodic fluctuations, especially during large public events or cultural promotion phases, where public enthusiasm rapidly increases, providing strong support for policy-making.

As for Guangdong’s network structure, it exhibits a more complex local clustering effect. Unlike Shanghai’s high aggregation and Beijing’s polycentricity, Guangdong’s network structure is characterized by distinct hierarchical and decentralized features. Particularly within specific social groups or regions, the spread of badminton attention shows a pronounced local concentration. This clustering effect is likely influenced by Guangdong’s regional cultural promotion, marketing strategies, and social activities. To some extent, Guangdong’s badminton network demonstrates a bottom-up expansion approach, where the cohesion and influence of local communities gradually promote the widespread dissemination of social behaviors. This network evolution path demonstrates how social activities, starting from local areas, eventually form broader impacts through collective efforts.

The aforementioned facts demonstrate the growing significance of critical nodes in network evolution. Figures 2 through 5 not only reflect the temporal changes in badminton attention but also reveal sharp structural shifts at specific time points within the network. These shifts are not merely chaotic fluctuations but rather represent the inherent nonlinear behavior of complex systems. Both complex network theory and dynamic systems theory (DST) suggest that the evolution of complex systems is often accompanied by the interaction between local nodes and global behaviors, ultimately leading to a transition from an orderly state to a chaotic state, and then returning to stability. The badminton attention networks in Shanghai, Beijing, and Guangdong have all experienced a transition from order to disorder, particularly under the influence of specific policy initiatives or social events, where the emergence of certain nodes exhibits increased volatility. For example, in Shanghai and Beijing, certain nodes experience rapid surges in attention, thereby driving the expansion of the entire network, while in Guangdong, a local explosive growth occurs within specific social groups. The appearance of these sudden nodes marks a “fracture” and restructuring of the network, demonstrating the system’s inherent self-organizing and nonlinear feedback mechanisms.

Critical nodes not only serve as turning points for the entire system but also act as “catalysts” when driving the network from a stable state into a chaotic one. These nodes are the transformative drivers within complex systems, triggering nonlinear reactions across the entire network within a short period, leading to global restructuring and adjustment. Through these nodes, public behavior is not merely a single response but, through interaction and feedback, continuously drives changes in social behavior patterns. The badminton attention networks in Shanghai, Beijing, Guangdong, and China as a whole all exhibit clear scale-free network characteristics. A scale-free network is one in which a few core nodes exist with high connectivity, allowing for rapid information spread and influencing the entire network. This characteristic is particularly prominent in the dissemination of badminton attention, especially in core cities like Shanghai and Beijing, where a few central nodes (such as social media platforms and the organizers of major events) play a decisive role in driving information propagation. The formation of a scale-free network allows information to spread rapidly across society and triggers the aggregation of public attention within a short time. In contrast, Guangdong’s badminton attention network displays a more decentralized characteristic. Although it also exhibits scale-free properties, a larger number of nodes are involved in the transmission and diffusion of information. This suggests that Guangdong’s social groups rely not only on the guidance of a few central nodes but are also driven by multiple local communities pushing the dissemination and participation of information. In this relatively decentralized network structure, the methods of information transmission are more diverse, exhibiting higher adaptability and resilience.

The national network of China displays a highly “core-periphery” structure, reflecting the hierarchical nature of the social network in disseminating badminton attention across the country. In this hierarchical structure, a few core areas with concentrated information (such as Shanghai and Beijing) dominate national badminton attention, while peripheral regions (such as the western areas) exhibit relatively independent network structures. This structure further reveals the dynamics of information transmission and its regional disparities. The variation in badminton attention reflects the adaptive responses of different social groups to policy, cultural promotion, and social activities. In Shanghai, information dissemination relies on the influence of a few core nodes; in Beijing, policy-driven initiatives and cultural diversity lead to a more balanced information spread; while in Guangdong, the promotion of local communities and localized activities demonstrates the localized and hierarchical nature of information dissemination. Policymakers should fully recognize these regional differences and implement strategies tailored to local conditions to promote badminton culture. In Shanghai and Beijing, policies should focus more on guiding core nodes and planning activities; in Guangdong, the emphasis should be on mobilizing local communities and fostering voluntary participation. At the national level, the government can further stimulate public attention and engagement by organizing large-scale badminton events and enhancing promotional efforts.

**Figure 10:**
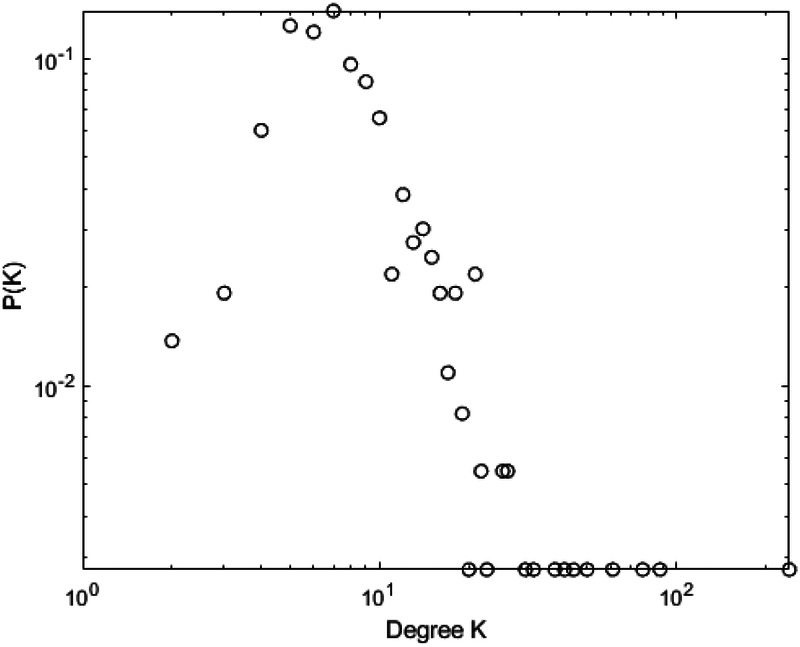
Degree distrbution of Shanghai.

**Figure 11:**
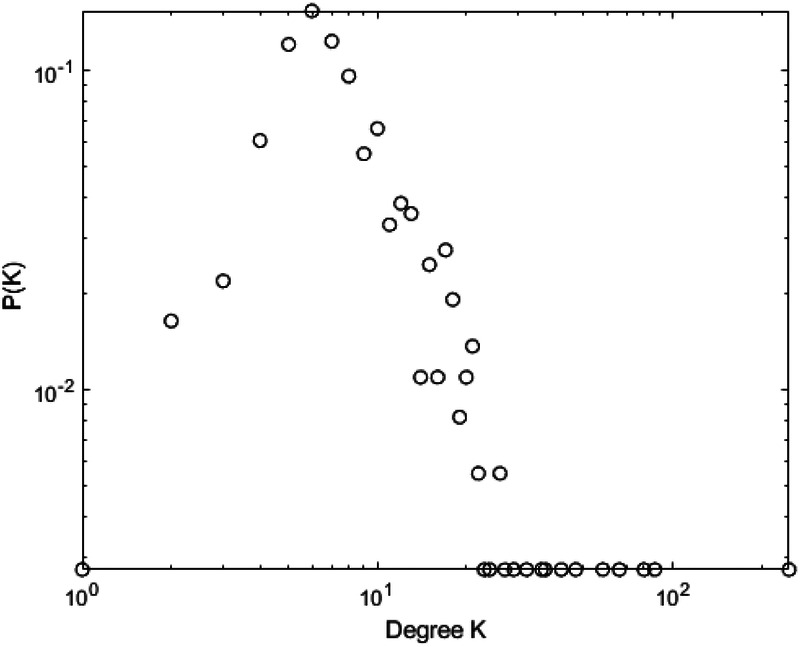
Degree distrbution of Beijing.

**Figure 12:**
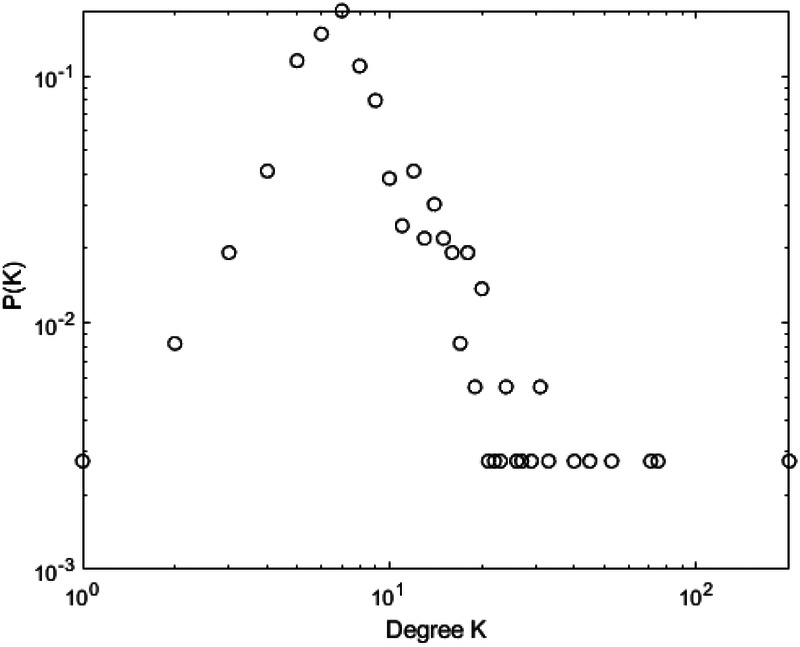
Degree distrbution of Guangdong.

**Figure 13:**
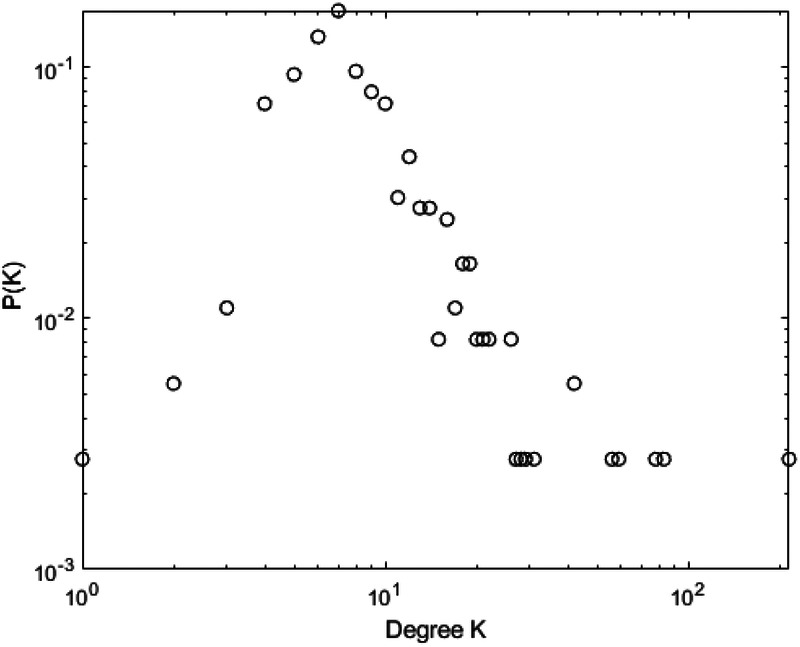
Degree distrbution of China.

Figures 10–13 illustrate the degree distribution characteristics of the badminton attention networks in Shanghai, Beijing, Guangdong, and China as a whole. Despite the degree distributions across these regions reflecting the structural features of scale-free networks—where a few nodes connect to a large number of other nodes and most nodes have relatively low degrees, exhibiting a power-law distribution—there are still subtle differences, particularly in the tail sections. These small discrepancies reflect variations in the efficiency, paths, and overall network structure evolution of information dissemination across different regions, revealing differences in the mechanisms of information spread, social group interactions, and the breadth and speed of dissemination.

Shanghai’s degree distribution shows a shorter tail, indicating that most nodes have low connectivity, with only a few nodes possessing high degrees. These core nodes play a dominant role in the network. The efficiency of information dissemination is high, and the propagation paths are relatively simple, with the clustering effect enabling rapid information spread through core nodes to other nodes in the network. Beijing’s degree distribution, in contrast, presents a longer tail, suggesting that the process of information dissemination is not solely reliant on a few core nodes, but rather more nodes contribute to information spread. This change implies that Beijing’s network structure is more decentralized and polycentric compared to Shanghai, with information dissemination no longer concentrated on a few core nodes but expanding to a wider group through the collective efforts of multiple social groups and platforms.

Guangdong’s degree distribution exhibits an even longer tail, indicating a network with more high-degree nodes, often located within local communities that drive localized information spread. Unlike Shanghai and Beijing, Guangdong’s network structure sees more nodes playing significant roles within local communities. While local communities can rapidly boost badminton attention, the spread of information across the overall network is slower due to the geographic and community-based limitations.

At the national level, core regions (such as Shanghai and Beijing) exhibit higher efficiency in information transmission, with shorter propagation paths, while peripheral regions show lower transmission efficiency, longer paths, and slower spread. Consequently, China’s network structure exhibits a classic “core-periphery” pattern, where nodes in core regions (e.g., Shanghai and Beijing) have higher connectivity, enabling information to spread quickly from these core nodes to a broader audience. However, information dissemination in peripheral regions is more restricted, leading to a lag in global transmission. These subtle differences have significant implications for the dynamics of information spread. Shanghai’s high clustering allows information to spread rapidly, particularly reaching a wider group in a short time. However, as the spread depends on a few core nodes, the process may lead to a certain uniformity of the information. In contrast, Beijing’s polycentric structure ensures greater diversity and reach of information, albeit with lower transmission efficiency, as information is distributed across more social groups. Guangdong’s local clustering effect reveals a regional characteristic of information dissemination; while local communities propel attention growth, the overall expansion speed is slower. The core-periphery structure of China as a whole illustrates regional differences in information dissemination, where core regions exhibit strong propagation capabilities, while peripheral regions experience delays, leading to an uneven distribution of national attention. The differences in network structures across regions reflect the interplay of various factors such as local cultural backgrounds, social group behaviors, and policy interventions. The feedback mechanisms and self-organizing behaviors in each region during the information spread process highlight their unique dissemination paths and mechanisms. These differences enhance our understanding of the dynamic changes in social behavior and information dissemination under different contextual backgrounds. In conclusion, despite similar degree distributions, significant differences exist in the social behavior patterns and information dissemination efficiency across regions. The evolution of badminton attention networks in Shanghai, Beijing, Guangdong, and China as a whole showcases the hierarchical, decentralized, and clustered nature of complex networks, revealing how information dissemination continuously evolves through nonlinear feedback mechanisms within different social groups, cultural settings, and policy environments.

**Figure 14:**
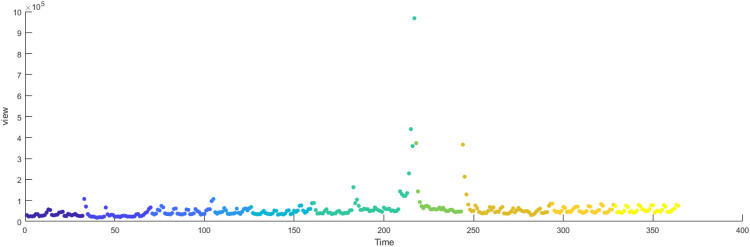
Evolutionary stage of Shanghai.

**Figure 15:**
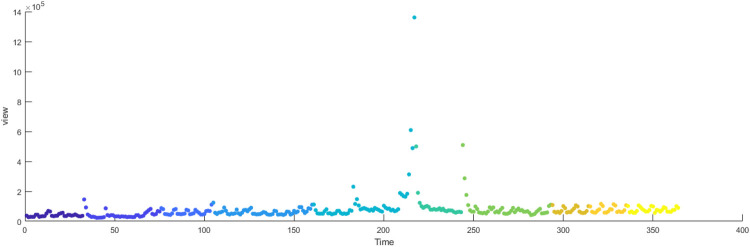
Evolutionary stage of Beijing.

**Figure 16:**
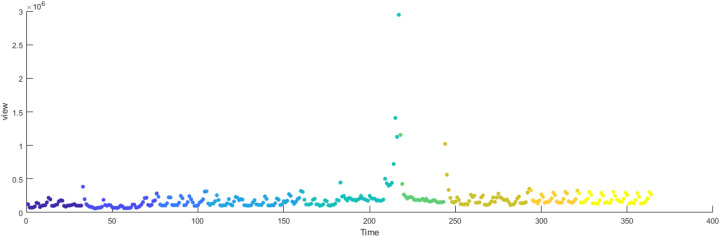
Evolutionary stage of Guangdong.

**Figure 17:**
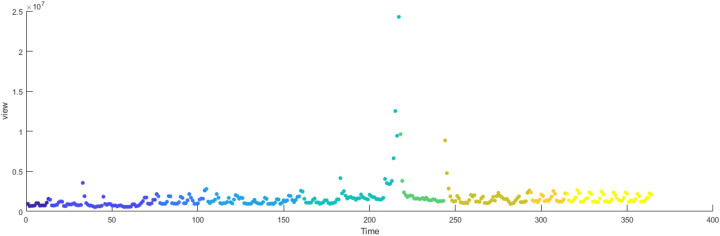
Evolutionary stage of China.

Figures 14–17 present the daily data segmentation of badminton attention from January 1, 2024, to December 29, 2024. The evolution of badminton attention can be divided into several distinct phases, especially around the 2024 Paris Olympics, where fluctuations in attention demonstrated significant stage-like characteristics. The data from Shanghai reveals a clear peak in attention from July to August 2024. This peak is closely related to the occurrence of the Paris Olympics, particularly in the weeks before and after the event, during which information dissemination surged sharply. Network analysis indicates that during this phase, Shanghai’s network structure experienced rapid aggregation, with a few high-connectivity nodes (such as social media platforms and sports news websites) quickly becoming the core channels for information dissemination. This process mirrors the “evolution from low-order to high-order organization” described in the study of complex systems in tourist destinations, where the rapid rise in badminton attention signals the swift growth of information spread, with the system transitioning from a low-order state to a relatively more ordered state.

In contrast, Beijing also experienced a rise in attention due to the Olympics, but compared to Shanghai, the changes were less dramatic, and the fluctuations in attention were more stable. This steady change suggests that Beijing’s badminton attention network is more decentralized, with multiple social platforms and groups collectively driving information spread, resulting in a more diversified propagation path. Although the speed of dissemination was slower, the cooperation between nodes within the network allowed attention to maintain steady growth over a longer period. Guangdong’s fluctuations, on the other hand, exhibited distinct regional characteristics, with dramatic changes occurring mainly during local events and social activities. Compared to other regions, Guangdong’s badminton attention was more reliant on local clusters to drive dissemination. While the spread of information was slower, its efficiency within localized areas was higher. This differs from the networks in Shanghai and Beijing, showcasing Guangdong’s network structure as more localized and self-organizing.

By applying the Horizontal Visibility Graph (HVG) algorithm to map these time series data, further insights into the small-world and scale-free characteristics of networks across regions can be revealed. These characteristics indicate that the dissemination of badminton attention exhibits notable nonlinear dynamics, where information spread is influenced not only by the dominant role of a few core nodes but also by self-organizing behaviors. External factors significantly contribute to the phase-like changes in badminton attention. As previously mentioned, important events, driven by external environments and the primary system, act as “key elements” in the system’s evolution. The Paris Olympics brought global attention to badminton, particularly in China, where badminton, as an important competitive sport, attracted significant attention from both viewers and media through relevant events and news coverage.

Before and after the Olympics, badminton attention in all regions experienced sharp increases, particularly around the event, where the speed and efficiency of information transmission significantly improved. This phenomenon reflects how external events can become pivotal factors in driving changes in network structure and information dissemination efficiency. However, after the conclusion of the Olympics, the enthusiasm for information dissemination gradually waned, and badminton attention in all regions stabilized. This process reveals the nonlinear dynamic behavior of information dissemination, where it is not continuous but rather alternates between the influence of external events and internal feedback mechanisms. The evolution of badminton attention networks also demonstrates significant self-organizing behavior. Shanghai, Beijing, and Guangdong all exhibited strong feedback mechanisms in their network structures, where information spread was driven by interactions between core nodes and multiple social groups, showing adaptive characteristics. Particularly in Guangdong, the interactions and feedback mechanisms between local communities played a crucial role in information dissemination.

## 5 Conclusion

This study employs Complex Network Theory, particularly the Horizontal Visibility Graph (HVG) algorithm, to transform daily Douyin search volume data for the keyword “badminton” (covering Beijing, Shanghai, Guangdong, and national-level data in China from January to December 2024) into a network structure. By integrating the Complex Adaptive System (CAS) theory and self-organization theory, we systematically analyzed the evolutionary dynamics and structural characteristics of the badminton attention complex system. The key findings are as follows: First, the badminton attention system exhibits nonlinear and event-sensitive dynamics, with significant surges in public attention driven by major events such as the 2024 Paris Olympics. Second, the attention network demonstrates small-world and approximately scale-free properties—Shanghai’s network is dominated by core nodes with high clustering, Beijing’s network shows polycentric decentralization, and Guangdong’s network features prominent local clustering, while the national network presents a clear “core-periphery” structure. Third, HVG analysis suggests potential chaotic tendencies in the system, though definitive proof of deterministic chaos requires further validation. These findings collectively reveal the complex evolutionary mechanisms of public attention toward badminton in the digital context.

Despite its contributions, this study has several limitations. First, the data is limited to the Douyin platform and Chinese provinces, lacking cross-country (e.g., Denmark, India, Malaysia) validation, making it impossible to generalize the findings to other contexts. Second, while the study identifies potential chaotic tendencies in the attention system, it lacks comprehensive verification using indicators such as Lyapunov exponents and surrogate data tests, resulting in insufficient evidence for claims of deterministic chaos. Third, the analysis focuses on macro-level network structures, with insufficient exploration of micro-level mechanisms (e.g., how individual user behavior and media algorithms influence attention evolution).

The research findings offer targeted guidance for formulating badminton promotion strategies, optimizing resource allocation, and enhancing the effectiveness of sports culture communication, with implications for multiple stakeholders including government sports departments, sports industry practitioners, and social media platforms.

In conclusion, the evolution of public attention toward badminton is a complex nonlinear process shaped by interactions between events, regions, and agents. This study provides a foundational understanding of this process, and future research addressing its limitations will further advance the scientific study of sports attention dynamics and inform more effective strategies for sports promotion and social behavior guidance.

## Data Availability

The raw data supporting the conclusions of this article will be made available by the authors, without undue reservation.
